# Mucormycosis in Different Clinical Settings: A Case Series

**DOI:** 10.7759/cureus.24419

**Published:** 2022-04-23

**Authors:** Tareq Esteak, Rabeea Shah, Maha Tosaddeque, Shameem Behram, Muhammad Nasir

**Affiliations:** 1 Neurology, National Institute of Neurosciences and Hospital, Dhaka, BGD; 2 Infectious Diseases, The Indus Hospital, Karachi, PAK; 3 Internal Medicine, Civil Hospital Karachi, Karachi, PAK; 4 Neurology, National Institue of Neurosciences and Hospital, Dhaka, BGD; 5 Infectious Disease, The Indus Hospital, Karachi, PAK; 6 General Practice, Pakistan Navy Station (PNS) Shifa Hospital, Karachi, PAK

**Keywords:** posaconazole, amphotericin, renal transplant, covid-19, diabetes, mucormycosis

## Abstract

Mucormycosis is a fungal infection that can be very destructive and often fatal. People most prone to this infection include those who are immunocompromised. Early diagnosis and treatment, along with addressing the risk factors, play a pivotal role in the management. Here, the authors are reporting three cases of immunocompromised patients. Among them, two had uncontrolled diabetes, and the third had a history of renal transplant and COVID-19 infection. All three cases are distinct anatomically; one is pulmonary, one is rhino-orbital-cerebral, and the last one is rhino-maxillary.

## Introduction

Mucormycosis is a rare but fatal fungal infection caused by mucor moulds, which are commonly found in the environment around us. It usually affects people with a suppressed immune system [1]. People with diabetes are one of the most vulnerable groups to be infected with it. In addition, people who are on prolonged corticosteroids and other immunosuppressive drugs are at risk as well [2]. The manifestations can be in multiple ways, with the rhino-orbital-cerebral form being one of the most common forms. The other common forms include rhino-orbital and rhino-maxillary forms [3]. Patients usually present with fever, headache, rhinorrhea, and occasionally multiple cranial nerve palsy [3]. This mould also tends to invade the vascular wall, causing damage to the tunica intima and, subsequently, ischemic damage to the affected tissue [4]. If untreated, it can bring devastating outcomes as the mortality rate is about 30-70%. So, the early detection of the condition and aggressive surgical and medical management are necessary to prevent a disastrous outcome [5].

## Case presentation

Case 1

**Figure 1:**
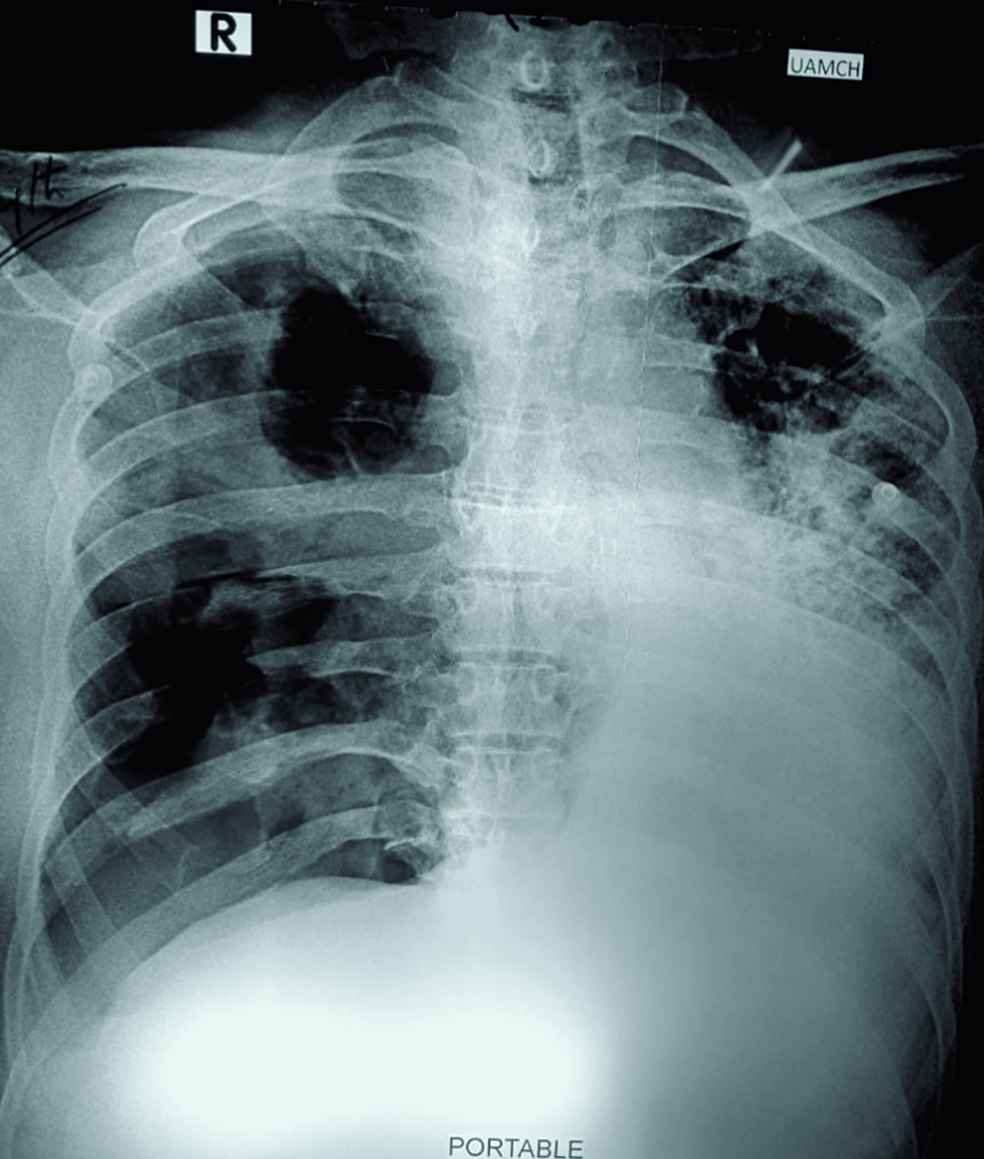
Portable chest X-ray revealing bilateral consolidation with left-sided effusion.

**Figure 2:**
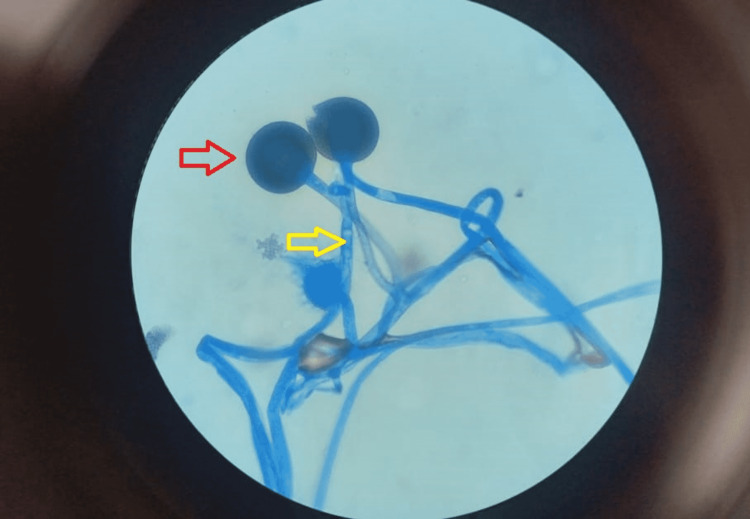
Photomicrograph shows lactophenol cotton blue-stained broad aseptate hyphae (yellow arrow) and the spherical structure (red arrow) called the sporangium (original magnification, ×100).

A 63-year-old man with a history of a renal transplant had been stable for six years. He was receiving maintenance immunosuppressive therapy consisting of tacrolimus and mycophenolate mofetil. The patient was admitted to the hospital eight days after the first onset of COVID-19 symptoms. He was clinically stable and was discharged after a couple of weeks, but later he developed severe respiratory distress and was admitted to the ICU. Subsequently, the investigations revealed bilateral consolidation (Figure 1) with pleural effusion on the left side of the chest radiograph. The pleural fluid analysis revealed Rhizopus microsporus by both microscopy (Figure 2) and culture of the pleural fluid. On the 12th day of admission, anti-fungal treatment with liposomal amphotericin B was introduced. The clinical status of the patient continued to deteriorate, and the patient expired on the 13th day of ICU admission. An autopsy was not performed.

**Figure 3:**
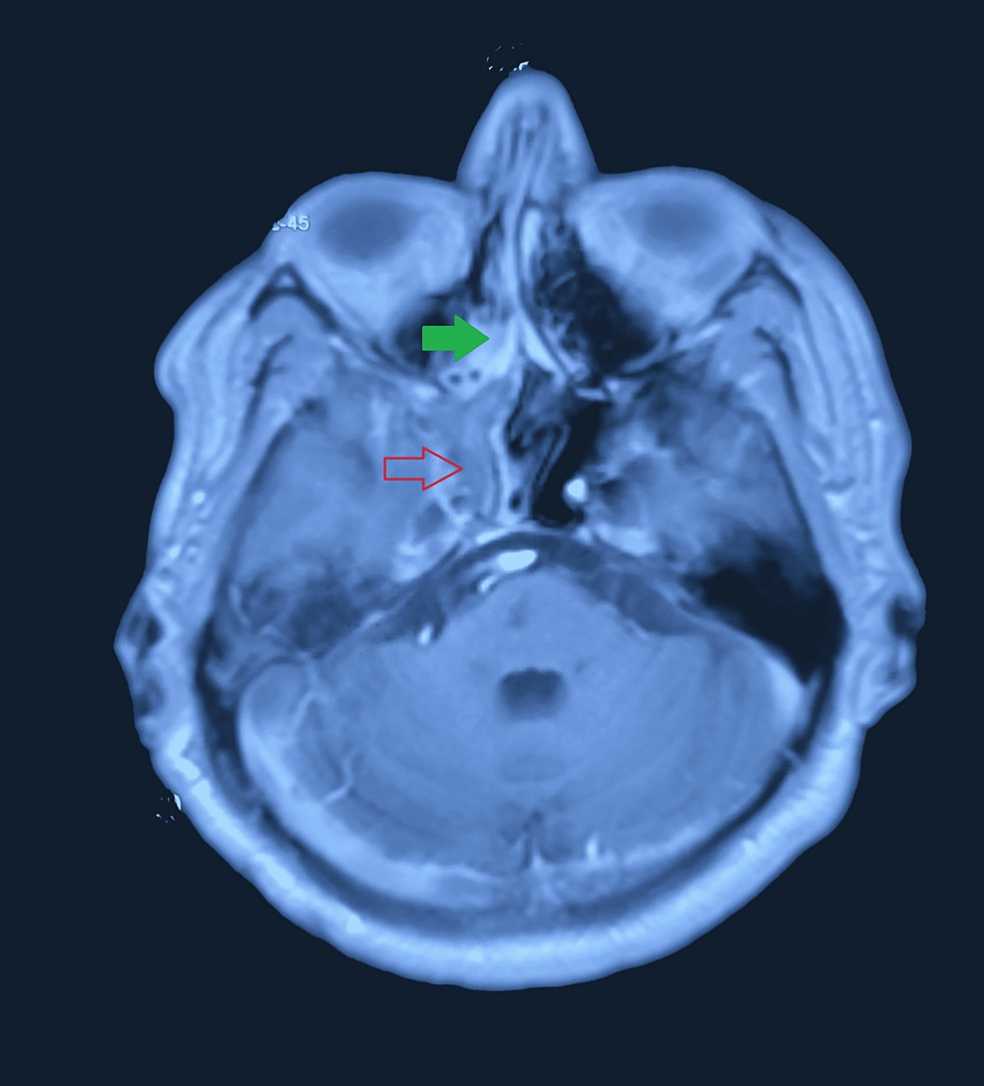
Contrast-enhanced MRI image showing mixed intensity lesion, with slight contrast enhancement involving the right maxillary and ethmoidal sinus (green arrow) extending into the right cavernous sinus region (red arrow). MRI: magnetic resonance imaging.

**Figure 4:**
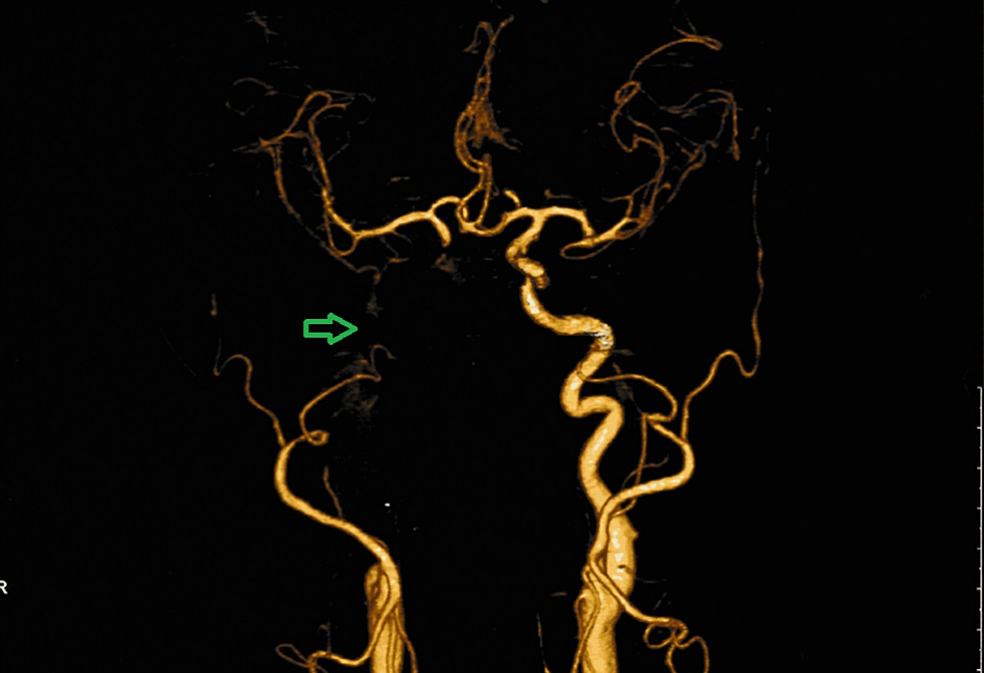
CT angiography cerebral and neck vessels showing non-visualized or completely occluded right internal carotid artery (green arrow) from its origin carotid bifurcation to supraclenoid part and a good collateral system. CT: computerized tomography.

**Figure 5:**
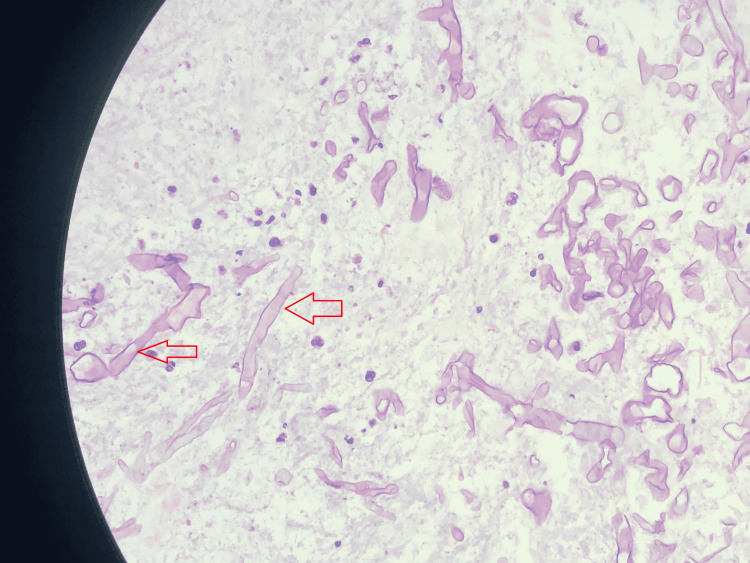
Photomicrograph shows H&E-stained tissue section showing aseptate irregular branching (red arrow) of fungal hyphae (original magnification, ×100). H&E: hematoxylin and eosin

Case 2

A 55-year-old diabetic and hypertensive male presented with a right-sided headache and drooping of the eyelid. The glycemic control was very poor, evidenced by an HbA1c of 8.3%. Afterwards, the neurological examination revealed right-sided ptosis with complete ophthalmoplegia. The MRI of the brain revealed (Figure 3) right maxillary, ethmoidal, and sphenoidal sinusitis with extension to the right cavernous sinus. In addition, the MR angiography showed complete occlusion of the right internal carotid artery (Figure 4). Afterwards, an endoscopic transnasal transsphenoidal surgical decompression was done, and the collected tissue was sent for histopathology. Histopathological examination revealed large areas of ribbon-like aseptate irregular branching fungal hyphae (Figure 5), suggestive of mucormycosis. Then, the patient was discharged home on oral Posaconazole. On follow-up after one month, his headache subsided completely, and his drooping and ophthalmoplegia significantly improved.

Case 3

**Figure 6:**
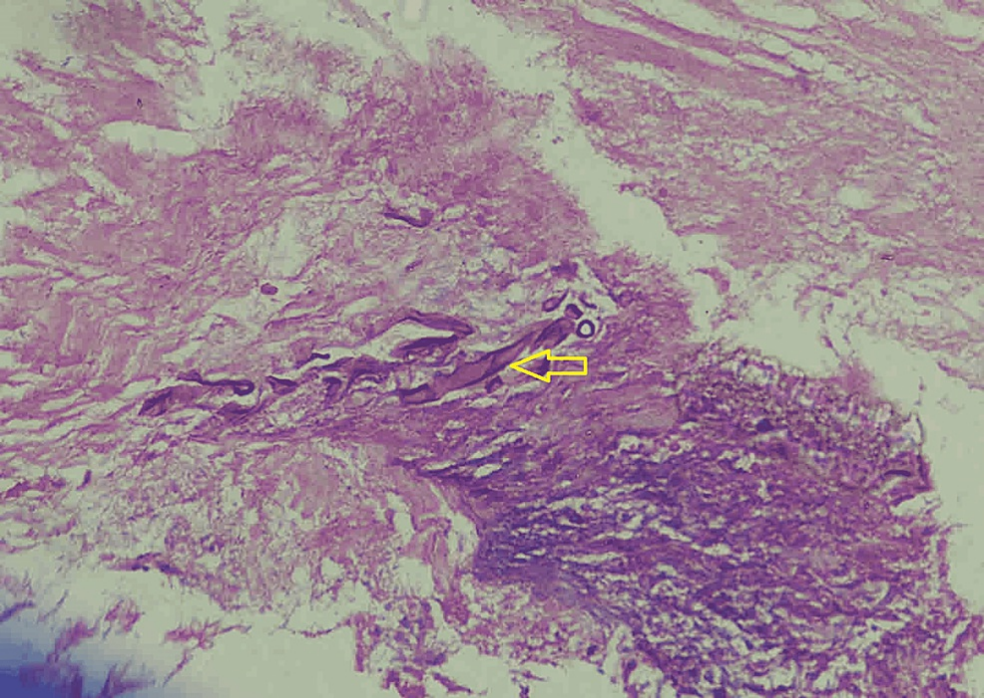
Photomicrograph shows H&E-stained tissue section showing aseptate irregular branching (yellow arrow) of fungal hyphae suggestive of mucormycosis (original magnification, x100). H&E: hematoxylin and eosin.

A 55-year-old diabetic male presented with a right-sided frontal headache, loosening of teeth, and blackish discolouration of the hard palate for one month. He was poorly compliant with his diabetic medications. The examination was consistent with partial right-sided ptosis and a black eschar on the hard palate, but other neurological examinations were grossly unremarkable. His blood workup suggested an Hba1c of 11% and raised inflammatory markers. The PNS and orbits CT scan reported enhancing, infiltrating, soft tissue density eroding maxillary, ethmoidal sinuses without orbital or intracranial extension. Endoscopic sinus surgery was done, and tissues were sent for histopathology. The histopathology revealed broad non-septate ribbon-like hyphae suggestive of mucormycosis (Figure 6). Antifungal therapy with IV amphotericin with 0.7 mg/kg IV daily was started. He continues to stay in treatment at the daycare facility of our hospital. Follow-up shows complete improvement of his partial ptosis, headaches and facial pain.

## Discussion

Mucormycosis is a rare filamentous and fatal fungal infection mostly encountered among immuno-suppressed patients [1]. They belong to the class of Zygomycetes with distinct patterns of clinical infection [4]. It can produce several serious clinical manifestations in different parts of the body when the person is immunocompromised [6].

The infection begins with the inhalation of the spores. In persons with an intact immune system, infection rarely develops because the fungal spores are phagocytized by macrophages [4]. From here, infection spreads to the paranasal sinuses and orbit. The fungus may gain access to the cavernous sinus and the brain parenchyma through the cribriform plate [3]. In diabetic patients, hyperglycemia causes pulmonary macrophage dysfunction. As a result, macrophages cannot engulf the inhaled fungal spores [7]. Moreover, hyperglycemia causes neutrophilic dysfunction, which encourages mycotic proliferation [3].

The initial symptoms of rhino-orbital-cerebral mucormycosis are consistent with either sinusitis or periorbital cellulitis, followed by the onset of conjunctival suffusion and blurry vision. Fever may be absent in up to half of the cases; leukocytosis is evident as long as the patient has functioning marrow [8]. The clinical manifestations of pulmonary mucormycosis are nonspecific. The most common symptoms include fever, cough, expectoration, and hemoptysis [2].

MRI is more sensitive than CT in detecting rhino-orbital-cerebral mucormycosis [9]. The chest radiology of pulmonary mucormycosis is mostly non-specific; an abnormality is found in >80% of patients. The findings include consolidation, cavitation, the air-crescent sign, the halo sign, the reversed halo sign, pulmonary nodules, and effusion. Similar to pulmonary aspergillosis, pulmonary mucormycosis is best detected with high-resolution CT to determine the extent of the disease [10].

The diagnosis is confirmed with histopathological identification of fungal structures and positive cultures. Histopathological findings include the detection of broad aseptate hyphae with right-angled branching in KOH mount and are pathognomonic of mucormycosis [5].

This management requires a multidisciplinary approach. Controlling the risk factors and stabilizing the patient’s general condition should be a priority [11]. In cases of rhino-orbital-cerebral mucormycosis, prompt initiation of systemic antifungal therapy, control of the underlying systemic condition, and aggressive radical debridement of sinuses are the keys to better outcomes [5]. In pulmonary mucormycosis, early Amphotericin B, along with surgical resection of the involved areas of the lung, is the mainstay of treatment [10]. Medical treatment with anti-fungals is essential and has a success rate of around 72% [1].

**Figure 7:**
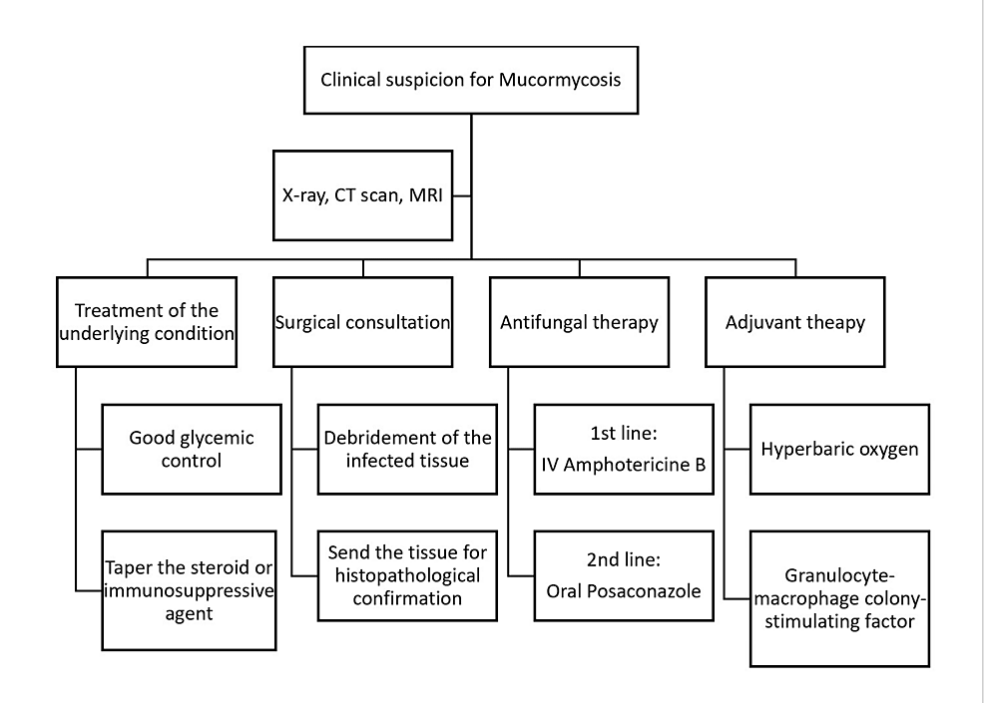
Proposed diagnostic and therapeutic approach for mucormycosis.

The choice of anti-fungal is liposomal amphotericin B, but it has a high risk of nephrotoxicity. Another alternative is oral posaconazole [1]. Other options for treatment include a hyperbaric oxygen chamber to provide oxygen to ischemic tissue so that neutrophils can effectively work and promote wound healing [12]. Finally, after managing all three cases of mucormycosis, we proposed a diagnostic and therapeutic approach that will help clinicians with prompt decision-making in cases of suspected mucormycosis (Figure 7).

## Conclusions

Mucormycosis is a destructive and serious infection with devastating complications. People with poorly controlled diabetes or with a compromised immune system are at a higher risk of getting infections. The key to proper management is early suspicion and diagnosis to start targeted treatment. Specific anti-fungals with surgical decompression might reduce mortality and morbidity. We would recommend that any physician who has suspected the condition should take prompt decisions towards confirming the diagnosis, thus decreasing the disastrous outcomes. The paper might serve as a platform for future studies on the diagnosis, treatment, and prognosis of mucormycosis.
